# Transcutaneous spinal stimulation in people with and without spinal cord injury: Effect of electrode placement and trains of stimulation on threshold intensity

**DOI:** 10.14814/phy2.15692

**Published:** 2023-06-02

**Authors:** Harrison T. Finn, Elizabeth A. Bye, Thomas G. Elphick, Claire L. Boswell‐Ruys, Simon C. Gandevia, Jane E. Butler, Martin E. Héroux

**Affiliations:** ^1^ Neuroscience Research Australia Randwick New South Wales Australia; ^2^ School of Biomedical Sciences University of New South Wales New South Wales Kensington Australia; ^3^ Prince of Wales Hospital Randwick New South Wales Australia; ^4^ School of Clinical Medicine University of New South Wales New South Wales Kensington Australia

**Keywords:** spinal cord injury, transcutaneous spinal stimulation

## Abstract

Transcutaneous spinal cord stimulation (TSS) is purported to improve motor function in people after spinal cord injury (SCI). However, several methodology aspects are yet to be explored. We investigated whether stimulation configuration affected the intensity needed to elicit spinally evoked motor responses (sEMR) in four lower limb muscles bilaterally. Also, since stimulation intensity for therapeutic TSS (i.e., trains of stimulation, typically delivered at 15–50 Hz) is sometimes based on the single‐pulse threshold intensity, we compared these two stimulation types. In non‐SCI participants (*n* = 9) and participants with a SCI (*n* = 9), three different electrode configurations (cathode–anode); L1‐midline (below the umbilicus), T11‐midline and L1‐ASIS (anterior superior iliac spine; non‐SCI only) were compared for the sEMR threshold intensity using single pulses or trains of stimulation which were recorded in the vastus medialis, medial hamstring, tibialis anterior, medial gastrocnemius muscles. In non‐SCI participants, the L1‐midline configuration showed lower sEMR thresholds compared to T11‐midline (*p* = 0.002) and L1‐ASIS (*p* < 0.001). There was no difference between T11‐midline and L1‐midline for participants with SCI (*p* = 0.245). Spinally evoked motor response thresholds were ~13% lower during trains of stimulation compared to single pulses in non‐SCI participants (*p* < 0.001), but not in participants with SCI (*p* = 0.101). With trains of stimulation, threshold intensities were slightly lower and the incidence of sEMR was considerably lower. Overall, stimulation threshold intensities were generally lower with the L1‐midline electrode configuration and is therefore preferred. While single‐pulse threshold intensities may overestimate threshold intensities for therapeutic TSS, tolerance to trains of stimulation will be the limiting factor in most cases.

## 1 INTRODUCTION

Transcutaneous spinal cord stimulation (TSS) has emerged as a complementary therapy to restore motor function in people with spinal cord injury (SCI; Megía‐García et al., 2020; Rahman et al., 2022; Shackleton et al., 2022). By stimulating afferent nerve fibers in the dorsal roots, TSS is thought to reflexively increase motoneurone excitability, which improves the ability of descending motor drive to recruit additional motoneurones below the level of the SCI (Barss et al., 2022; Mayr et al., 2016; Taccola et al., 2018). Despite its growing popularity, some fundamental aspects of TSS methodology have yet to be explored systematically.

To be effective, TSS should preferentially target muscles that play a key role in the motor task being trained. Ideally, stimulating electrodes should be positioned so that the administered electrical current uniformly excites the multiple motoneurone pools innervating these muscles. However, despite the relatively diffuse current induced by TSS, dorsal roots closer to the cathode electrode tend to experience higher currents, which leads to uneven dispersion of current across the motoneurone pools (Rattay et al., 2000). For example, proximal leg muscles appear to be recruited preferentially when the cathode is positioned over T11–T12, while distal muscles appear to be recruited preferentially when the cathode is positioned over L1–L2 (Krenn et al., 2013; Militskova et al., 2020; Roy et al., 2012; Salchow‐Hömmen et al., 2019; Sayenko et al., 2015). Much less is known about the choice of anode location choice in determining how stimulation interacts with the target motoneurone pools. Some studies regard the choice as inconsequential and choose the one reported as most comfortable by the participant (e.g., Zaaya et al., 2021). However preliminary evidence suggests that different anode placements (e.g., abdomen vs. the anterior superior iliac spines (ASIS)) can lead to differential changes in excitability between motoneurone pools (Masugi et al., 2017), presumably by altering the flow of current through the body (Danner et al., 2011; Ladenbauer et al., 2010).

All else being equal, an electrode configuration that requires lower stimulation intensities would be preferred as it would likely be more comfortable. Conversely, if different electrode configurations resulted in a similar ability to excite the target motoneurone pools, they could be used interchangeably and thus accommodate clinical contraindications such as supra‐pubic catheters or surgical implants. Thus, a detailed comparison of the most common electrode configurations used to target lower limb muscles provides information that would inform the use of TSS with locomotor training in people with SCI.

Another key aspect of TSS methodology that varies between studies is the method for determining stimulation intensity. In general, absolute measures of intensity are of limited use. Several factors such as electrode size (Skiadopoulos et al., 2022), anatomical differences (Binder et al., 2021), waveform (Dalrymple et al., 2023; Manson et al., 2020; Sayenko et al., 2019), and frequency (Sayenko et al., 2019) will alter the efficacy of a given current. Consequently, a train of stimulation at a given intensity (in milliamps (mA)) can induce slight paresthesia in one person but widespread motoneurone recruitment in another person. For these reasons, the determination of TSS stimulation intensity is frequently linked to achieving a physiological outcome.

Common approaches to setting stimulation intensity use either subjective criteria such as participant tolerance, paresthesia or limb movement with trains of stimulation, generally 15–50 Hz (Bedi & Arumugam, 2016; Estes et al., 2021; Gad et al., 2017; Gerasimenko et al., 2015; Gorodnichev et al., 2012; Hofstoetter et al., 2013; Hofstoetter, Krenn, et al., 2015; McHugh et al., 2020; Sayenko et al., 2019), or neurophysiological criteria such as determining the lowest single‐pulse stimulation intensity required to evoke a spinally evoked motor response (sEMR) and then setting stimulation intensity around that (Hofstoetter et al., 2020; Meyer et al., 2020; Minassian et al., 2016; Siu et al., 2022; Sutor et al., 2022; Zaaya et al., 2021). Subjective criteria, such as tolerance and paresthesia, are flawed due to individual differences in the severity of a person's injury and the associated sensory deficits, hypersensitivity, anatomical features, and neuropathic pain. Similarly, the ability to detect limb movement induced by TSS is limited by the sensitivity of the measure used, variability across individuals and antagonist muscle activity. Applying these subjective criteria among participants may cause variability in the level of engagement of the neural circuits activated by TSS across participants.

In contrast, neurophysiology criteria, such as the lowest intensity required to elicit an sEMR, can serve as an objective criterion by determining the minimum level of TSS intensity needed to cause motoneurone firing—presumably by engaging the proposed neurophysiology mechanisms underpinning therapeutic TSS (Angeli et al., 2014; Dy et al., 2010; Gad et al., 2017; Gerasimenko et al., 2015; Sayenko et al., 2019). This enables therapeutic stimulation intensity to be set relative to this threshold and standardized between participants. However, no consensus on what amount of stimulation relative to threshold intensity has been established: some stimulate at threshold intensity (Siu et al., 2022), while others stimulate above (Minassian et al., 2016; Shapkova et al., 2020; Zaaya et al., 2021) or below threshold intensity (Hofstoetter et al., 2020; Hofstoetter, Krenn, et al., 2015; Momeni et al., 2022; Samejima et al., 2022). Since relevant muscles may have different threshold intensities for evoking sEMR, setting the therapeutic intensity based on the first recruited muscle will result in all other muscles being stimulated at subthreshold intensities; the opposite problem exists if stimulation intensity is set based on the threshold intensity of the last recruited muscle. To limit the influence of these two extreme conditions, TSS should be administered with an electrode configuration that yields relatively similar threshold stimulation intensities across the targeted muscles.

A critical aspect of TSS methodology that has received little attention is whether threshold intensities to evoke sEMR determined with single pulses are comparable to those obtained with trains of stimulation (15–50 Hz). Trains of stimulation repetitively activate axons, which will change axonal excitability (Burke et al., 2001), increase the activation of polysynaptic circuits via interneurons (Jilge, Minassian, Rattay, & Dimitrijevic, 2004; Minassian et al., 2004), or induce post‐activation depression of the Ia synapse (Hofstoetter, Danner, et al., 2015; Vargas Luna et al., 2021; see also Hultborn et al., 1996). Based on results from studies investigating paired TSS pulses (e.g., 50 ms interpulse intervals; Hofstoetter et al., 2018; Minassian et al., 2007), we would expect the stimulation intensity required to evoke a sEMR would be higher with trains of stimulation compared to a single pulse.

Therefore, in a group of non‐SCI participants and a group of participants with a SCI, this study aimed to: (1) document the stimulus intensities required to evoke sEMR in four key lower limb locomotor muscles using three TSS electrode configurations, and (2) compare the stimulation intensity required to evoke sEMR with single pulses and trains of stimulation. We hypothesized that the electrode configuration would impact the stimulation intensity required to evoke sEMR in the target muscles, and that threshold intensities would be increased in trains of stimulation compared to single pulses.

## 2 METHODS

### 2.1 Participants

Twenty‐five participants were recruited for the study: 14 without a SCI (non‐SCI) and 11 with a complete or incomplete SCI (Table 1). Participants were excluded if they were pregnant or had: electrical implants, spinal hardware between T11–L4, a history of fainting, a malignancy, or a neurological disease or injury (other than SCI). Two participants with a SCI withdrew: one due to hypertension on arrival that increased when tilted into a standing position and another due to an inability to tolerate TSS at intensities high enough to elicit any sEMR. Four non‐SCI participants withdrew from the study due to postural hypotension during the experiment. Also, data from one non‐SCI participant were excluded due to poor quality EMG recordings. Thus, data were analyzed from nine non‐SCI participants (Table 2) and nine participants with a SCI (Table 1). Participants were not selected based on any prior knowledge of whether sEMR could be elicited in lower limb muscles.

**TABLE 1:** Characteristics of participants with a spinal cord injury and the incidence of spinally evoked motor response.

Participant ID	Neurological level of injury	AIS	Time since injury (years)	Age	BMI	sEMR incidence T11‐midline		sEMR incidence L1‐midline	
						Single	Train	Single	Train
SCI_10	C3	C	3	63	22.0	2/8	0/8	8/8	1/8
SCI_9	C3	C	5	36	18.0	8/8	0/8	8/8	0/8
SCI_7	C3	D	5	26	18.6	6/8	6/8	6/8	6/8
SCI_6	C7	C	4	43	29.7	3/8	0/8	5/8	0/8
SCI_5	T4	B	4	41	26.6	8/8	8/8	8/8	8/8
SCI_3	T5	C	1	44	25.4	6/8	5/8	6/8	5/8
SCI_8	T7	A	17	56	24.3	7/8	0/8	7/8	5/8
SCI_4	T10	C	2	35	32.4	3/8	0/8	0/8	0/8
SCI_1	T12	C	9	30	19.5	2/8	0/8	4/8	1/8
Total incidence						45/72	19/72	52/72	26/72
Mean (SD)				42 (11.9)	24.1 (5.0)				

*Note*: Denominator represents the total number of muscles recorded.

Abbreviations: AIS, American spinal injuries association (ASIA) impairment scale; BMI, body mass index; sEMR, spinally evoked motor response.

**TABLE 2:** Characteristics of non‐SCI participants and the incidence of spinally evoked motor response.

Participant ID	Age	BMI	sEMR incidence L1‐ASIS		sEMR incidence T11‐midline		sEMR incidence L1‐midline	
			Single	Train	Single	Train	Single	Train
01	45	24.5	8/8	3/8	8/8	0/8	8/8	0/8
03	42	23.7	8/8	0/8	8/8	8/8	8/8	8/8
04	32	25.5	8/8	8/8	8/8	7/8	8/8	6/8
05	29	22.5	7/8	0/8	8/8	0/8	8/8	0/8
06	28	23.2	7/8	0/8	7/8	5/8	7/8	0/8
07	32	20.2	8/8	3/8	8/8	2/8	8/8	2/8
08	33	24.7	5/8	2/8	8/8	2/8	8/8	6/8
10	26	24.9	7/8	0/8	6/8	4/8	8/8	0/8
12	38	18	7/8	0/8	8/8	0/8	8/8	0/8
Total incidence			65/72	16/72	69/72	28/72	71/72	22/72
Mean (SD)	33 (6.0)	23 (2.0)						

*Note*: Denominator represents the total number of muscles recorded.

Abbreviation: sEMR, spinally evoked motor response.

### 2.2 Experimental design

#### 2.2.1 Stimulation

**FIGURE 1:**
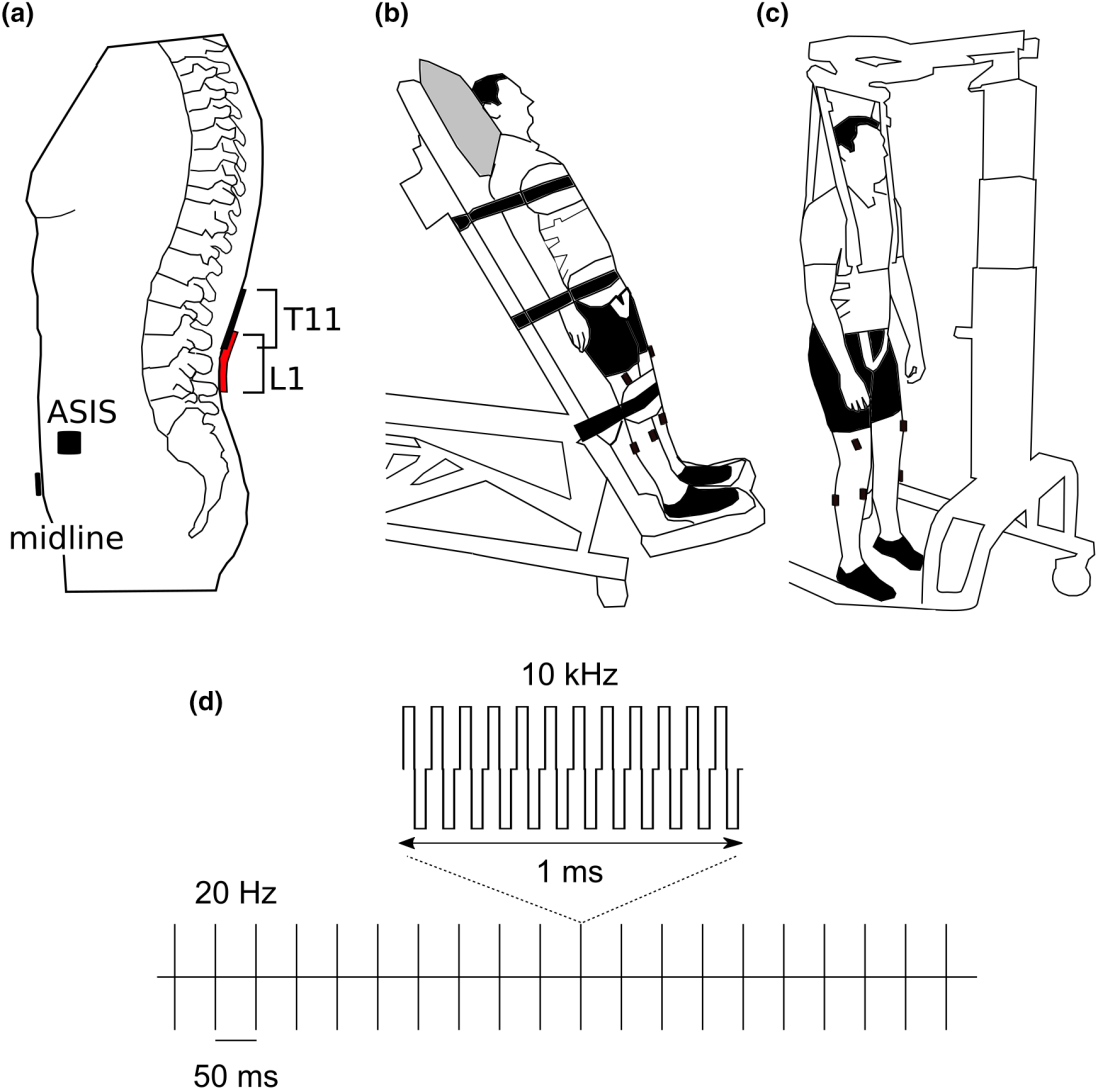
Experimental setup for transcutaneous spinal stimulation. (a) Shows the two different electrode configurations. The cathode electrode (10 × 5 cm) was placed vertically along the midline at T11 (black) or L1 (red). The anode electrode was placed horizontally along the midline of the abdomen (10 × 5 cm) approximately 3 cm above the pubic symphysis or on both anterior superior iliac spines (ASIS; 5 × 5 cm). (b) Testing position for participants with spinal cord injury: tilt table inclined to 55°. Electromyography (EMG) electrodes were placed bilaterally over the vastus medialis, tibialis anterior, medial gastrocnemius, and medial hamstring muscles. (c) Testing position for non‐SCI participants: suspended in body‐weight support harness. The same EMG set‐up was used. (d) Schematic representation of the stimulation waveform used for single pulses and trains of stimulation. Each pulse contained 10, 40 μs biphasic pulses at 10 kHz; trains were delivered at 20 Hz.

Three electrode configurations were tested: T11‐midline, L1‐midline, and L1‐ASIS (Figure 1a). For the T11‐midline configuration, the cathode was a 10 × 5 cm rectangular, adhesive electrode (Axelgaard Manufacturing Co. Ltd) placed longitudinally over the spinal column along the midline with the upper margin of the electrode between the T11 and T12 spinous processes, and the anode was a 10 × 5 cm electrode placed horizontally in the midline ~3 cm above the upper border or the pubic bone. Larger electrodes were chosen as we aimed to excite several motor pools that exist over several spinal segments (Sharrard, 1964). Larger electrodes that span many spinal segments may also reduce the recruitment threshold for sEMR (Gerasimenko et al., 2015; Skiadopoulos et al., 2022).

For the L1‐midline configuration, the upper margin of the cathode was placed between the L1 and L2 spinous processes longitudinally along the midline; the anode was in the same position as for the T11‐midline configuration. For the L1‐ASIS configuration, the cathode was in the same position as for the L1‐midline configuration and the anode was a pair of 5 × 5 cm electrodes placed bilaterally over each ASIS.

Data were collected from the non‐SCI participants first, and all three configurations were tested. Due to the lower incidence of sEMR and the higher stimulation intensities required to elicit these responses with the L1‐ASIS configuration (see Section 3), it was not tested in participants with a SCI to reduce the experimental burden on these individuals.

Stimulation was delivered via a constant current stimulator (DS8R, Digitimer Ltd, Hertfordshire, England) driven by a custom‐built microcontroller circuit based on an Arduino Due (32‐bit ARM core, Arduino, Italy). The circuit produced a 1 ms waveform that consisted of ten 40 μs biphasic pulses delivered at 10 kHz. This waveform could be sent as a single pulse or a train of pulses at 20 Hz (Figure 1c). Stimulation frequencies used during trains of stimulation vary widely (Rehman et al., 2023), with frequencies from 5 to 100 Hz demonstrating varying degrees of benefits to locomotion movements, often including large interpersonal variability (Gorodnichev et al., 2012; Krenn et al., 2023). We chose 20 Hz because (1) in pilot testing 20 Hz it was perceived as more comfortable compared to 30 Hz, (2) the interstimulus interval was 50 ms which provides an adequate window to capture the entire sEMR, and (3) to be in line with our ongoing clinical trial (Bye et al., 2022).

#### 2.2.2 Electromyography

Surface electromyography (EMG) was recorded with a wireless system (Trigno Avanti Sensor, Delsys Inc.). Recordings were made from the right and left vastus medialis (VM), medial hamstring (Ham), medial gastrocnemius (MG), and tibialis anterior (TA) muscles. The skin was cleaned and lightly abraded with an alcohol wipe and gauze before the EMG electrodes were applied (bipolar configuration, 1 cm inter‐electrode spacing). EMG signals were filtered (bandpass 30–500 Hz) and amplified (×3000; 1902; Cambridge Electronics Design) prior to being digitized at 2 kHz with a 16‐bit CED DAQ‐card and Spike2 software (1401‐Power; Cambridge Electronics Design).

#### 2.2.3 Participant position

We were interested in testing sEMR in a position that was similar to what would be used in locomotion intervention studies. Therefore, participants were tested in a vertical position. Non‐SCI participants were suspended with 100% body weight support in a harness from an assistive mobility device (LiteGait 400S, LiteGait; Figure 1b). This ensured lower limb muscles were relaxed when spinal stimulation was administered. Participants were lowered every few trials. Despite these rest periods, four non‐SCI participants withdrew from the study as they experienced hypotension related to the position and harness. As a result, we altered the set‐up for participants with a SCI. Participants with a SCI were positioned on a tilt table inclined at 55° (Figure 1b). The trunk, hips and knees were firmly strapped down to eliminate weight bearing and associated lower limb muscle activity. Participants with a SCI were offered to be lowered every few minutes; these breaks were rarely required. To limit the differences between non‐SCI and participants with a SCI, participants with a SCI also wore the harness used to suspend non‐SCI participants from the body weight support device. The straps were tightened in the same way in both groups, ensuring the compression on the anode and cathode electrodes was similar.

### 2.3 Experimental protocol

The lowest stimulation intensity required to evoke sEMR (i.e., threshold intensity) was determined for each muscle. Due to the large number of muscles recorded in this study, threshold intensities were determined as stimulation intensity was increased in steps of 10 mA.

**FIGURE 2:**
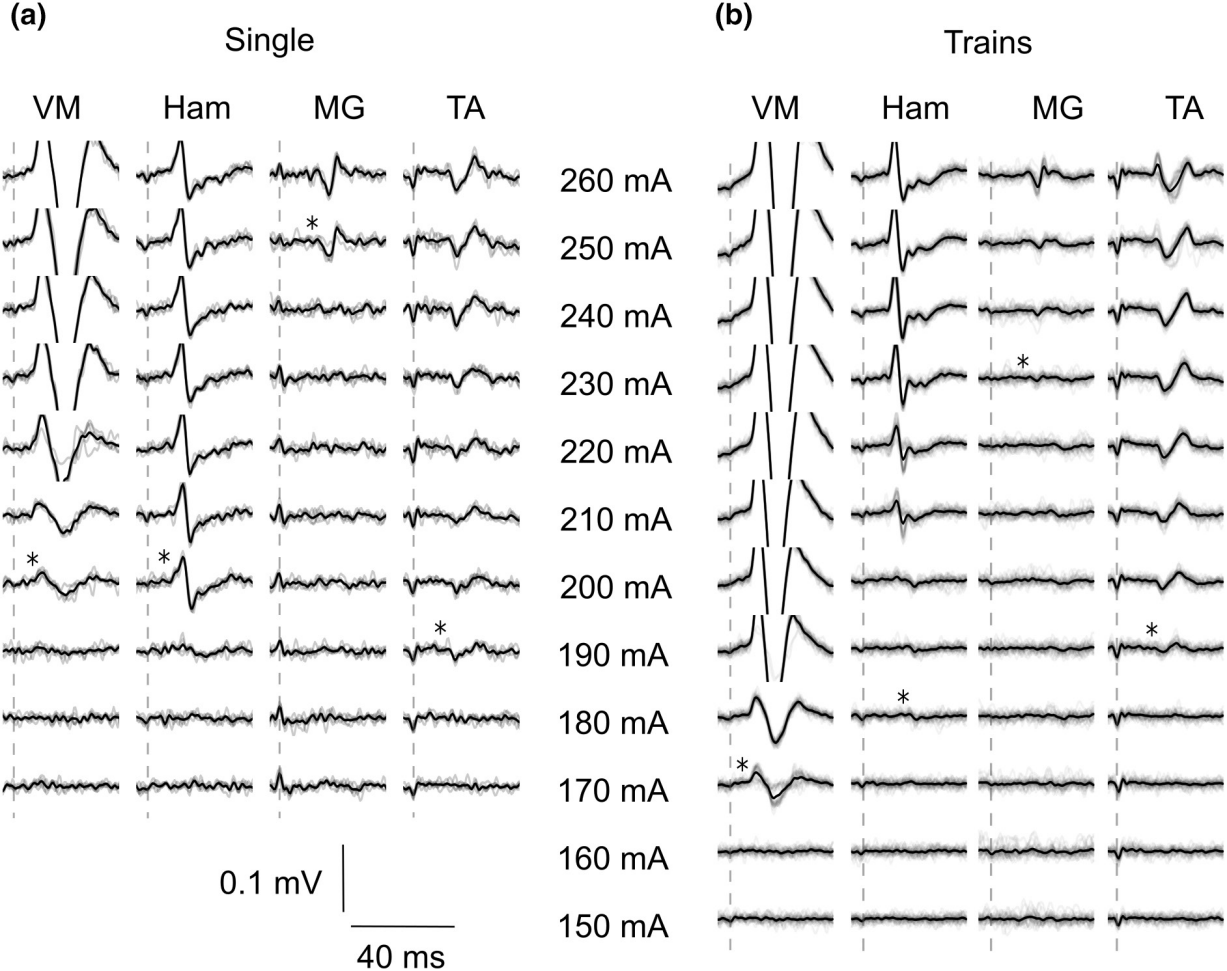
Representative spinally evoked motor response (sEMR) for a participant with a spinal cord injury for the single pulses (a) and trains of stimulation (b) for the four muscles on the right side. The electrode configuration used was L1‐midline. Individual traces (gray; single pulses *n* = 5; trains of stimulation *n* = 30) and the average waveform (black) are shown. The * indicates the threshold intensity to evoke sEMR for each muscle (see Section 2). The vertical line indicates the timing of the single‐pulse transcutaneous spinal stimulation. Ham, medial hamstring muscle; MG, medial gastrocnemius muscle; TA, tibialis anterior muscle; VM, Vastus medialis muscle.

First, single‐pulse stimulation was delivered at increasing stimulation intensities until a sEMR was evoked in any lower limb muscle. This intensity was then decreased by 20 mA, rounded down to the nearest multiple of 10 mA. Starting at this intensity, five pulses (5 s intervals) were delivered. Stimulation intensity was increased in steps of 10 mA until: (1) sEMR were elicited in all muscles, (2) participants indicated they could not tolerate the stimulation, or (3) further increases in stimulation intensity did not elicit sEMR in new muscles; this occurred in a few participants, even with increases in stimulation intensity as large as 100 mA (Figure 2a).

For trains of stimulation, intensity started at 0 mA. Stimulation intensity was increased in steps of 10 mA (kept constant for 2–3 s) until: (1) sEMR were present in all lower limb muscles, or (2) participants indicated they could not tolerate the trains of stimuli (Figure 2b).

Electrode configurations (Figure 1a) were tested in random order. Participants were asked to remain relaxed throughout the experiment, and muscle activity recordings were continuously monitored.

### 2.4 Data analysis

All data were analyzed with custom scripts written in the Python programming language (version 3.7, Python Software Foundation). For each stimulation intensity, pulse triggered‐averages (−50 ms to 100 ms; *n* = 5 for single pulses, *n* = 30 for trains of stimulation) were computed, which were then root‐mean‐squared (RMS) filtered (7 ms window). An algorithm determined the presence of a sEMR, based on peak rmsEMG amplitude following stimulation (4–50 ms) three times higher than pre‐stimulus rmsEMG amplitude (mean −50 to −4 ms). The threshold intensity to elicit a sEMR was identified for each muscle and for each electrode configuration. A researcher blinded to the participant group and electrode configuration verified the accuracy of selected sEMR threshold intensities (68.3% agreement). Most errors were within 1 step (i.e., 10 mA); larger errors were usually due to large stimulus artifacts in the proximal muscles of some participants. When there was disagreement between the algorithm and the researcher, the threshold intensity identified by the researcher was used.

For each electrode configuration, the threshold intensities of each muscle were ranked in ascending order for each participant. Muscles that had the same threshold intensity were given equal rank and subsequent ranks were adjusted; for example, if two muscles were recruited at a stimulation intensity of 200 mA, and these were the first recruited muscles, each would get a rank of 1, and the next recruited muscle would get a rank of 3. The latency and amplitude of trigger‐averaged sEMR at threshold intensity were also determined for each muscle.

### 2.5 Statistical analysis

For the non‐SCI and SCI groups, chi‐squared tests were used to compare the incidence of sEMR across muscles, across electrode configurations, and across stimulation types (i.e., single pulses, trains of stimulation).

Linear mixed models were used to compare the highest stimulation intensity reached in the experiment. For each participant group, a model was created to compare across electrode configurations and stimulation types. The fixed effects were electrode configuration (T11‐midline, L1‐ASIS (non‐SCI only), L1‐midline) and stimulation type (single pulses, trains of stimulation); interaction terms were included in the model. Next, a model was created to compare the highest stimulation intensity reached between participant groups, and across electrode configurations (T11‐midline, L1‐midline) and stimulation types (single pulses, trains of stimulation); participant group, electrode configuration, and stimulation type were all fixed effects. Two interaction terms were included in the model: participant group by electrode configuration and participant group by stimulation type.

Linear mixed models were also used to compare sEMR threshold intensity, ranks of muscle sEMR, sEMR latencies, and sEMR amplitudes. For each participant group, three models were created. The first two models were the same but were applied to data from each of the stimulation types (single pulses, trains of stimulation). Fixed effects were: side (left, right), electrode configuration (T11‐midline, L1‐ASIS (non‐SCI only), L1‐midline), and muscle group (VM, Ham, TA, MG). The interaction term of muscle group by electrode configuration was included in the model. The final model added stimulation type as a fixed factor and included interaction terms of stimulation type by electrode configuration and stimulation type and muscle group.

The incidence of sEMR was low during trains of stimulation, which could have been the result of participants not tolerating sufficiently high‐stimulation intensities during trains of stimulation. Thus, for each participant group, paired *t*‐tests were used to compare the sEMR threshold intensity for single pulses to the highest intensity reached during trains of stimulation for muscles that did not show a sEMR with the trains of stimulation.

A final model was created to compare across participant groups, and this was only for the single‐pulse stimulation type. The fixed effects were participant group (non‐SCI, SCI) electrode configuration (T11‐midline, L1‐midline) and muscle group (VM, Ham, TA, MG). For this analysis, the main effect of participant group and the interactions that included participant groups were of interest: participant group by electrode configuration and participant group by muscle group.

For all linear mixed models, participants were a random factor (intercept). When a significant fixed or interaction effect was found, pairwise Bonferroni corrected comparisons were made, and contrast estimates and 95% confidence intervals (95% CI) are reported. Figures display means and standard deviations. Statistical analyses were conducted with SPSS 26 software (v26.0.0.1, IBM Corporation). The threshold for all statistical analyses was *p* < 0.05.

## 3 RESULTS

Nine participants in each group completed the study. Individual demographic data for participants with and without SCI are presented in Tables 1 and 2. The latency and amplitude of sEMR evoked in each muscle by single pulses and trains of stimulation for both groups of participants are included in Data S1 and S2.

### 3.1 Incidence of spinally evoked motor responses with single‐pulse stimulation

Across electrode configurations, the incidence of sEMR with single‐pulse stimulation was higher in non‐SCI participants (95%, 205/216 possible sEMR) compared to participants with a SCI (67%, 97/144, *p* < 0.001). The lower incidence of sEMR in participants with a SCI was likely not due to differences in stimulation intensity because the highest intensity used did not differ between groups (*F*_(1, 33)_ = 0.624, *p* = 0.435).

**FIGURE 3:**
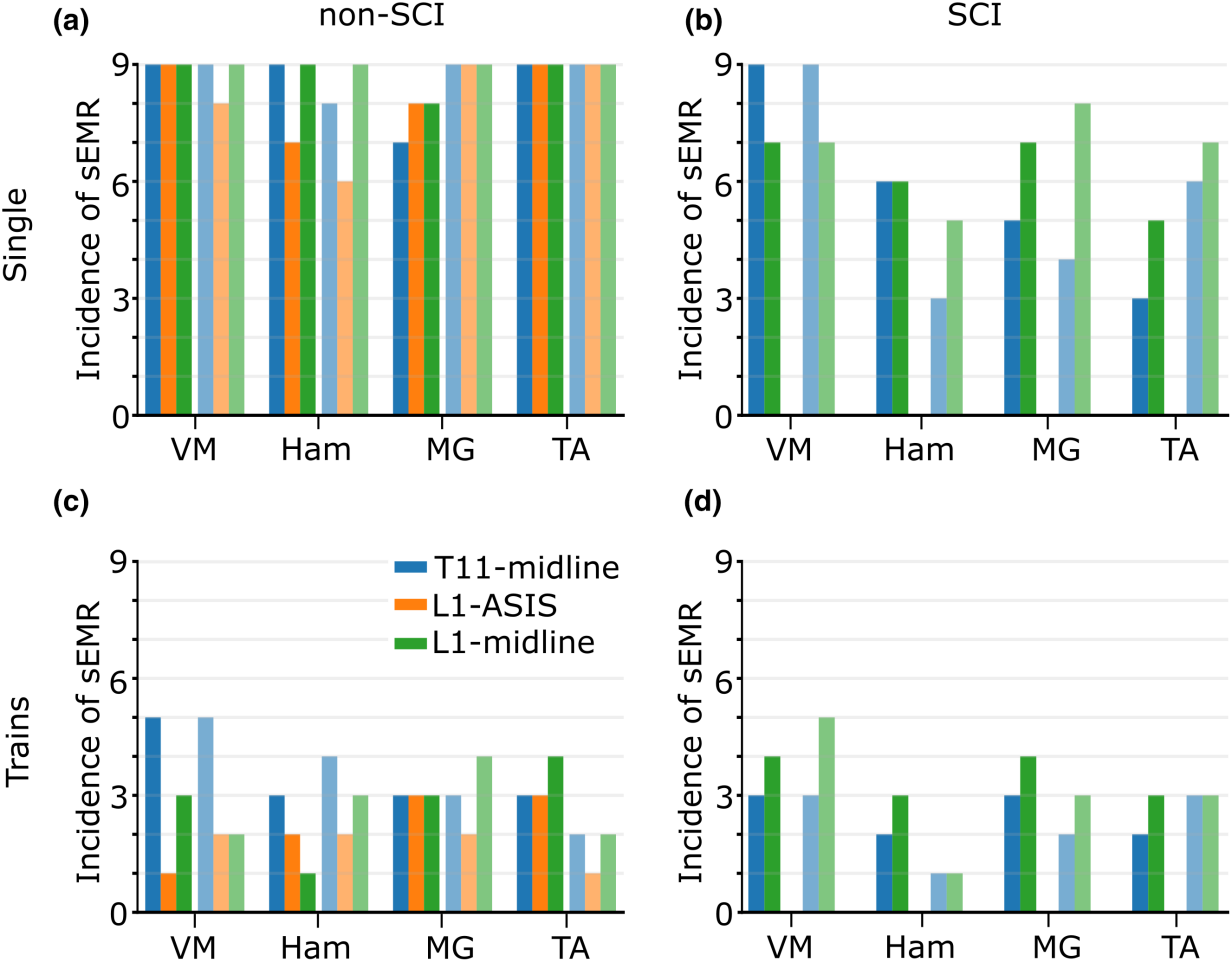
Incidence of a spinally evoked motor response (sEMR) for each muscle group and electrode configuration (blue: T11‐midline; orange: L1‐ASIS; green: L1‐midline). Solid bars to the left of each muscle label represent muscles on the left side of the body; lighter bars to the right of each muscle label represent muscles on the right side of the body. The incidence of sEMR in non‐spinal cord injury participants (*n* = 9) is shown in panels (a; single pulses) and (c; trains of stimulation). The incidence of sEMR in participants with spinal cord injury (*n* = 9) is shown in panels (b; single pulses) and (d; trains of stimulation). The L1‐ASIS electrode configuration was not tested in participants with a spinal cord injury. ASIS, anterior superior iliac spines; Ham, medial hamstring muscle; MG, medial gastrocnemius muscle; SCI, spinal cord injury; TA, tibialis anterior muscle; VM, vastus medialis muscle.

In non‐SCI participants (Figure 3a; Table 1), the incidence of sEMR was 99% for L1‐midline, 96% for T11‐midline and 90% for L1‐ASIS. The incidence was lower for L1‐ASIS than L1‐midline (*p* = 0.03). The highest stimulation intensity achieved during threshold intensity testing differed across electrode configurations: 272 mA (SD 34) for L1‐midline, 288 mA (SD 34) for T11‐midline and 323 mA (SD 60) for L1‐ASIS (*F*_(2, 16)_ = 13.121, *p* < 0.001). Specifically, it was greater for L1‐ASIS than T11‐midline (36 mA [6 to 64] (mean [95% CI]), *p* = 0.017) and L1‐midline (51 mA [19 to 82], *p* = 0.002), whereas it was similar between L1‐midline and T11‐midline (−16 mA [−40 to 9.49], *p* = 0.206).

In participants with a SCI, the incidence of sEMR was 72% for L1‐midline and 62% for T11‐midline (*p* = 0.28; Figure 3c). There was no clear pattern in terms of incidence (i.e., the ability to elicit sEMR) and the level of severity of the injury (Table 1). The highest stimulation intensity achieved was similar across electrode configurations: 300 mA (SD 56) for L1‐midline and 295 mA (SD 62) for T11‐midline (*F*_(1, 8)_ = 1.407, *p* = 0.270).

### 3.2 Incidence of spinally evoked motor responses with trains of stimulation

Across electrode configurations, the incidence of sEMR with trains of stimulation was lower for trains of stimulation across electrode configurations (*p* < 0.001, Figure 3). There was little or no difference in incidence between non‐SCI participants (30%, 66/216) and participants with a SCI (31%, 45/144; *p* = 0.89). The highest intensity of stimulation achieved for the two configurations common to both participant groups (i.e., T11‐midline, L1‐midline) was similar between groups (−58 mA [−122 to 6] non‐SCI participants vs. participants with a SCI; *F*_(1, 32)_ = 3.472, *p* = 0.072).

In non‐SCI participants (Figure 3b; Table 1), the incidence of sEMR was 39% for T11‐midline, 31% for L1‐midline, and 22% for L1‐ASIS (*p* = 0.03: T11‐midline vs. L1‐ASIS). Also, the highest stimulation intensity achieved during threshold intensity testing differed across electrode configurations: 154 mA (SD 63) for L1‐midline, 151 mA (SD 62) for T11‐midline, and 186 mA (SD 55) for L1‐ASIS (*F*_(2, 23)_ = 6.673, *p* = 0.008). It was greater for L1‐ASIS compared to T11‐midline (34 mA [0.39 to 68], *p* = 0.047) and L1‐midline (36 mA [0.41 to 72], *p* = 0.047), whereas it was similar for T11‐midline and L1‐midline (2 mA [−26 to 30], *p* = 0.868). The lower incidence of sEMR during trains of stimulation was likely due to the lower stimulation intensities reached. Across electrode configurations, the highest intensity used was lower for trains of stimulation than single pulses (−130 mA [−150 to −110]; *F*_(1, 43)_ = 190.861, *p* < 0.001).

In participants with a SCI (Figure 3d), the incidence of sEMR was 36% for L1‐midline and 26% for T11‐midline (*p* = 0.21). The highest stimulation intensity achieved was similar between electrode configurations: 225 mA (SD 68) for L1‐midline and 198 mA (SD 77) for T11‐midline (*F*_(1, 7)_ = 3.490, *p* = 0.103). Again, the lower incidence of sEMR during trains of stimulation was likely due to the lower stimulation intensities reached (−86 mA [−116 to −55]; *F*_(1, 25)_ = 40.803, *p* < 0.001).

### 3.3 Spinally evoked motor response thresholds

#### 3.3.1 Single pulses

**FIGURE 4:**
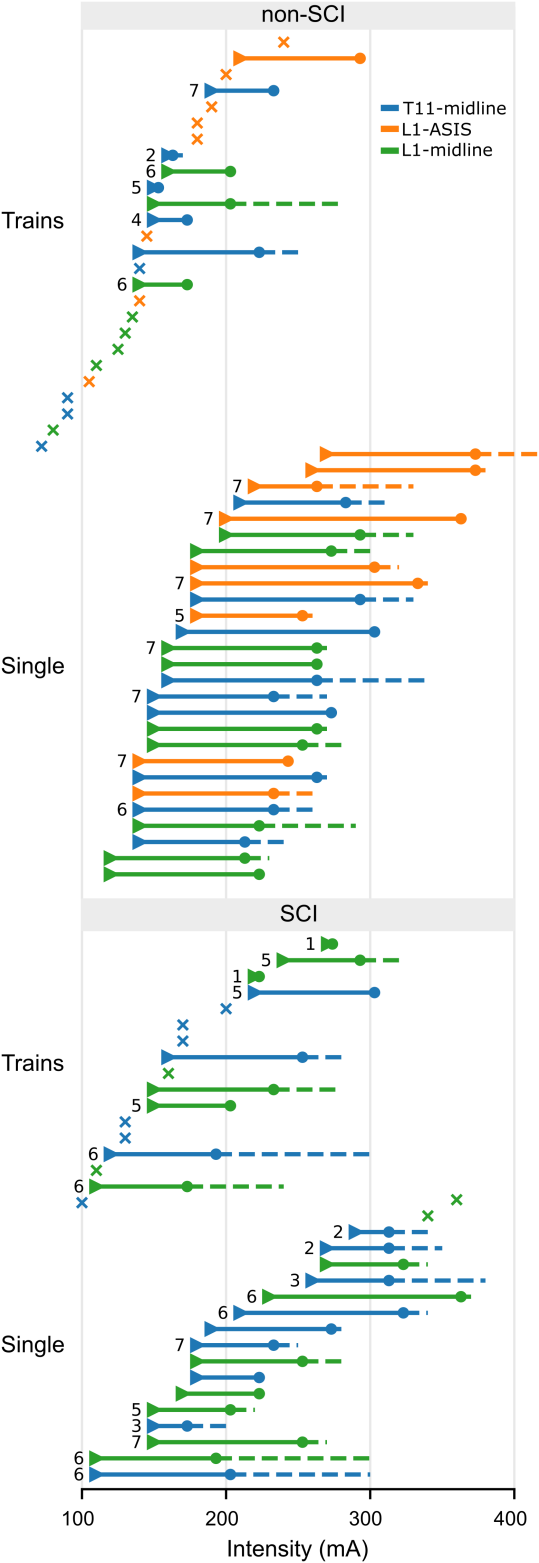
Intensities at which the first (triangle) and last (circle) spinally evoked motor responses (sEMR) were evoked for each participant for each electrode configuration (blue: T11‐midline; orange: L1‐ASIS; green: L1‐midline). If sEMR were not evoked in all 8 muscles (bilateral vastus medialis, tibialis anterior, medial gastrocnemius and medial hamstring muscles), a number is included to the left of the triangle to indicate the total number of muscles that showed sEMR. The dashed line represents increases in stimulation intensity without recruiting additional sEMR. The ‘x’ symbol indicates the intensity reached in cases where no sEMR was evoked for that experimental condition. ASIS, anterior superior iliac spine; SCI, spinal cord injury.

In non‐SCI participants, the stimulation intensities required to evoke sEMR in right and left lower limb muscles were similar (*F*_(1, 192)_ = 1.436, *p* = 0.234; Figure 4a). Moreover, there was no interaction between muscle group and electrode configuration (*F*_(6, 192)_ = 1.842 *p* = 0.093). However, there was an effect of electrode configuration on stimulation threshold intensity (*F*_(2, 192)_ = 61.249, *p* < 0.001; Figure 4b): L1‐midline was lower than T11‐midline (−16 mA [−26 to −6], *p* = 0.002) and L1‐ASIS (−57 mA [−69 to −44], *p* < 0.001), while T11‐midline was lower than L1‐ASIS (−40 mA [−52 to −28], *p* < 0.001).

There was also an effect of muscle group on stimulation threshold intensity (*F*_(3, 192)_ = 35.375, *p* < 0.001; Figure 4b). The threshold intensity for VM was lower compared to MG (−17 mA [−31 to −2], *p* = 0.016) and Ham (−57 mA [−73 to −40], *p* < 0.001), but not TA (−12 mA [−25 to 1], *p* = 0.087). Conversely, Ham had a higher threshold intensity compared to TA (45 mA [29 to −51] *p* < 0.001) and MG (40 mA [24 to 55], *p* < 0.001). Threshold intensity was similar between TA and MG (−5 mA [−17 to 6], *p* = 0.402).

For each participant, the threshold intensity of each muscle was ranked based on the order of recruitment. In non‐SCI participants, there was an effect of muscle group on rank (*F*_(3, 193)_ = 66.44, *p* < 0.001) with the average ranks for VM being lower (i.e., recruited earlier) than MG (−1.2 [−2.15 to −0.28], *p* < 0.006) and Ham (−3.79 [−4.57 to −3.01], *p* < 0.001), but not TA (−0.49 [−1.14 to 0.16], *p* = 0.139). While there was no main effect of electrode configuration (*F*_(2, 193)_ = 0.936, *p* = 0.394), there was an interaction between muscle group and electrode configuration (*F*_(6, 193)_ = 2.739, *p* = 0.014; Figure 4c). For VM, the average rank was lower for T11‐midline compared to L1‐midline (−1.2 [−2.3 to −0.07], *p* = 0.033). For Ham, the average rank was lower for L1‐ASIS compared to T11‐midline (−1.3 [−2.45 to −0.17], *p* = 0.018).

Similar to non‐SCI participants, the stimulus intensity required to evoke sEMR in right and left lower limb muscles in participants with a SCI was similar (*F*_(1, 80)_ = 2.975, *p* = 0.088; Figure 4d). Spinally evoked motor response threshold intensities were also similar between muscle groups (*F*_(3, 80)_ = 0.337, *p* = 0.798), and electrode configurations (*F*_(1, 80)_ = 1. 373, *p* = 0.245), with no interaction (*F*_(3, 80)_ = 0.797, *p* = 0.499; Figure 4e).

In participants with a SCI, there was an effect of muscle group on the rank order of muscle recruitment (*F*_(3, 20)_ = 3.768, *p* = 0.027). Specifically, VM had a lower rank than TA (−1.29 [−2.56 to −0.02], *p* = 0.046) but not Ham (−1.37 [−2.75 to 0], *p* = 0.051) or MG (−0.39 [−1.49 to 0.7], *p* = 0.76). There was also an interaction between muscle group and electrode configuration (*F*_(3,20)_ = 6.612, *p* = 0.003; Figure 4f). For VM, the average rank was lower for T11‐midline compared to L1‐midline (−1.5 [−2.5 to −0.59], *p* = 0.005). For MG, the average rank was lower for L1‐midline compared to T11‐midline (−2.66 [−4.38 to −0.94], *p* = 0.007).

#### 3.3.2 Trains of stimulation

**FIGURE 5:**
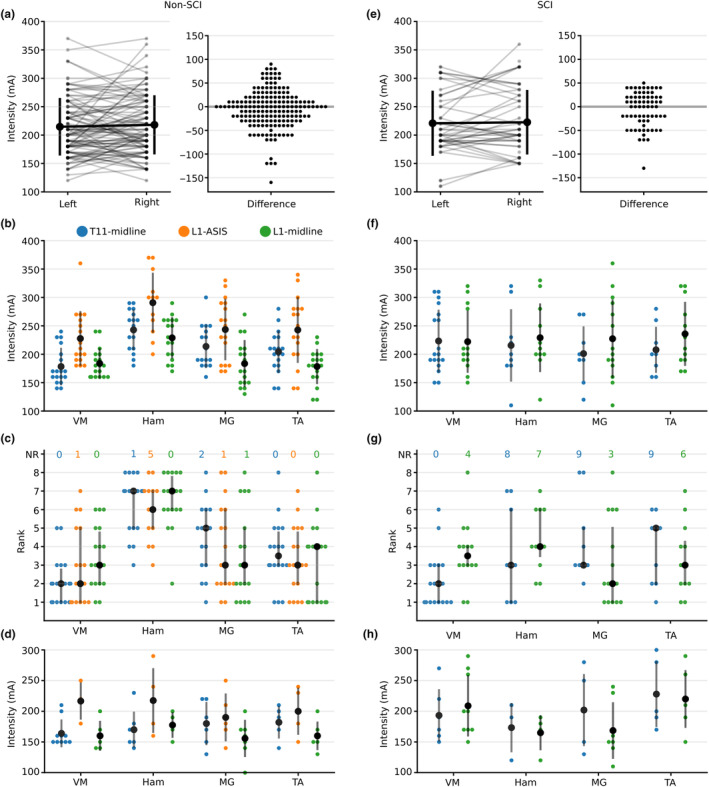
The stimulation threshold intensity (mA) to elicit a spinally evoked motor response (sEMR). Results for non‐spinal cord injury (non‐SCI) participants are in the left panels (a–d) and those for participants with a SCI are in the right panels (e–h). (a, e) Stimulation threshold intensity for single pulses in the left and right legs (all muscles pooled; left sub‐panel) and differences between the right and left legs (right sub‐panel). (b, f) Stimulation threshold intensity in each muscle group (left and right muscles pooled) for each electrode configuration (Blue, T11‐midline; Orange, L1‐ASIS; Green, L1‐midline). (c, g) Rank (out of 8, 1 = first recruited) of the stimulation threshold intensity across muscles (left and right muscles pooled). The count of no response (NR) for each muscle and electrode configuration is displayed at the top of the panel. Black circles indicate group means; error bars standard deviation for all figures except (c) and (g) which display the median and interquartile range. (d, h) Stimulation threshold intensity to evoke sEMR with trains of stimulation for each muscle group (left and right sides pooled), and electrode configurations. ASIS, anterior superior iliac spine; Ham, medial hamstring muscle; MG, medial gastrocnemius muscle; TA, tibialis anterior muscle; VM, vastus medialis muscle.

For trains of stimulation in non‐SCI participants, there was an effect of electrode configuration on threshold intensity (*F*_(2, 27)_ = 25.778, *p* < 0.001; Figure 5a): L1‐midline was lower than L1‐ASIS (−42.9 mA [−63.9 to −21.9], *p* < 0.001), but similar to T11‐midline (−13.8 mA [−27 to 0.19], *p* = 0.053), whereas T11‐midline was lower than L1‐ASIS (−29.4 mA [−48. 5 to −9.7], *p* = 0.003). There was no effect of muscle group (*F*_(3, 26)_ = 0.664, *p* = 0.582), nor was there an interaction (*F*_(6, 26)_ = 0.496, *p* = 0.806).

For trains of stimulation in participants with a SCI, there was no effect of electrode configuration (*F*_(1, 32)_ = 1.421, *p* = 0.242) or muscle group (*F*_(3, 14)_ = 0.812, *p* = 0.497) on threshold intensity (Figure 5b). There was also no interaction (*F*_(3, 29)_ = 2.296, *p* = 0.097).

### 3.4 Difference in sEMR threshold intensity between non‐SCI participants and participants with a SCI

For single pulses, there was no main effect of participant group on sEMR threshold intensities (*F*_(1, 227)_ = 2.292, *p* = 0.131). There was an interaction between participant group and electrode configuration (*F*_(1, 227)_ = 10.188, *p* = 0.002). Nevertheless, for the contrast of interest, sEMR threshold intensities were similar between groups for L1‐midline (−43 mA [−87.83 to 1.19], *p* = 0.056, non‐SCI participants vs. participants with a SCI) and T11‐midline (−19.5 mA [−64.11 to 25.08], *p* = 0.368, non‐SCI participants vs. participants with a SCI). There was also a participant group by muscle group interaction (*F*_(3, 211)_ = 8.028, *p* < 0.001). sEMR threshold intensity was higher for VM in participants with a SCI (49 mA [4 to 95], *p* = 0.033).

### 3.5 Difference in sEMR threshold intensity between single pulses and trains of stimulation

**FIGURE 6:**
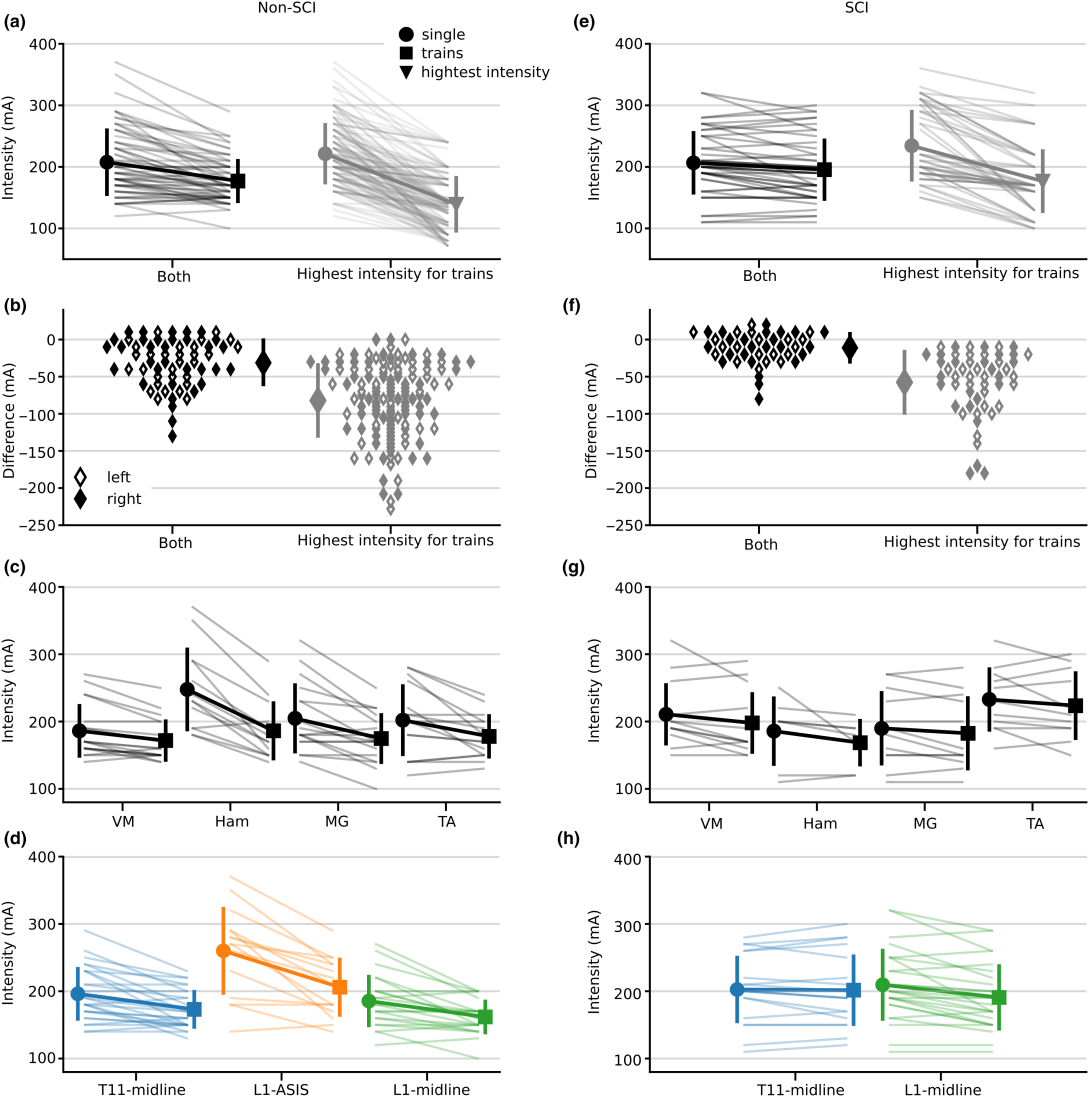
Comparison of stimulation threshold intensity to elicit spinally evoked motor response (sEMR) with single pulses and trains of stimulation. Results for non‐spinal cord injury (non‐SCI) participants are in the left panels (a–d) and those for participants with a SCI are in the right panels (e–h). (a, e) Results, pooled across muscles and sides, where single pulses (●) and trains of stimulation (■) both elicited a sEMR (‘Both’); if trains of stimulation did not elicit sEMR, the highest intensity reached during trains of stimulation (▲) was compared to the stimulation threshold intensity for single pulses (‘Highest intensity for trains’). (b, f) Differences between stimulation threshold intensity for single pulses and trains stimulation of the data depicted in panels A and E: right (♦) and left (◊) side muscles. (c, g) Comparison of stimulation threshold intensity to elicit a sEMR with single pulse pulses (●) and trains of stimulation (■) for each muscle group (left and right sides combined). (d, h) Comparison of threshold stimulation intensity required to elicit a sEMR with single pulses (●) and trains of stimulation (■) for each electrode configuration (Blue, T11‐midline; Orange, L1‐ASIS; Green, L1‐midline). Large symbols and error bars are means and standard deviations. ASIS, anterior superior iliac spine; Ham, medial hamstring muscle; MG, medial gastrocnemius muscle; TA, tibialis anterior muscle; VM, vastus medialis muscle.

In non‐SCI participants, sEMR were evoked by both single pulses and trains of stimulation in 63/216 muscles across the three electrode configurations. For those muscles with sEMR, threshold intensity was lower for trains of stimulation compared to single pulses ((*F*_(1, 112)_ = 62.647, *p* < 0.001); −35 mA [−44 to −26]; Figure 6a, left panel). That is, threshold intensity was, on average, 13% lower for trains of stimulation. There was a muscle group by stimulation type interaction (*F*_(3, 112)_ = 4.778, *p* = 0.004; Figure 6c). For the contrasts of interest, which compared stimulation type for each muscle, all muscles had lower threshold intensities with trains of stimulation (Ham: −64 mA [−83 to −45], *p* < 0.001, MG: −31 mA [−47 to −14], *p* < 0.001; TA: −26 mA [−43 to −6], *p* = 0.004; VM: −19 mA [−36 to −3], *p* = 0.021). There was also an electrode configuration by stimulation type interaction (*F*_(2, 112)_ = 4.319, *p* = 0.016; Figure 6d). For the contrasts of interest, which compared stimulation type for each electrode configuration, all three configurations had lower threshold intensities with trains of stimulation (L1‐ASIS: −55 mA CI [−73 to −37], *p* < 0.001; L1‐midline: −26 mA [−40 to −11], *p* = 0.001; T11‐midline: −25 mA [−38 to −11], *p* < 0.001).

To better understand why sEMR were sometimes not evoked in lower limb muscles with trains of stimulation in non‐SCI participants (*n* = 134/216 across all three electrode configurations), we compared the threshold intensity for single pulses (for the muscle in question) to the highest intensity reached with trains of stimulation for those muscles without sEMR (Figure 6a right panel, gray data). This highlighted that the highest intensity reached with trains of stimulation was 83 mA [75 to 89], 36% lower than the threshold intensity for single pulses (*F*_(1, 254)_ = 513.338, *p* < 0.001).

In participants with a SCI, sEMR were evoked by both single pulses and trains of stimulation in 44/144 muscles across the two electrode configurations. Threshold intensity was similar between single pulses and trains of stimulation (*F*_(1, 76)_ = 2.762, *p* = 0.101; Figure 6e,f left panels), with threshold intensities for trains of stimulation being 95% (SD 9) of those for single pulses. There was no muscle group by type of stimulation interaction (*F*_(3, 76)_ = 0.102, *p* = 0.959; Figure 6g) or electrode configuration by type of stimulation interaction (*F*_(1, 76)_ = 2.209, *p* = 0.141; Figure 6h).

Similar to the analysis carried out for non‐SCI participants, we sought to understand why sEMR were sometimes not observed with trains of stimulation in participants with a SCI (*n* = 53/144 across the two electrode configurations). We again compared threshold intensity for single pulses (for the muscle in question) to the highest intensity reached with trains of stimulation (Figure 6e,f right panel, gray data) and found that the highest intensity with trains of stimulation was 54 mA [44 to 64]; 24% lower than the threshold intensity for single pulses (*F*_(1, 93)_ = 114.806, *p* < 0.001).

## 4 DISCUSSION

The present study is unique because it investigated systematically how sEMR are evoked in lower‐limb muscles in non‐SCI participants and participants with a SCI with different electrode configurations, and with trains of stimulation compared to single pulses. Electrode configuration influenced the threshold intensity required to evoke sEMR in four lower limb muscles. In non‐SCI participants, threshold intensities were lower in the T11‐midline and L1‐midline electrode configuration compared to the L1‐ASIS configuration, in which the incidence of sEMR was also the lowest. In participants with a SCI, there was no difference in threshold intensity and sEMR incidence between the T11‐midline and L1‐midline configurations. Where it was possible to evoke lower limb sEMR with trains of stimulation, the threshold intensity was reduced by 13% compared to single pulses for non‐SCI participants and was similar in participants with an SCI. However, the incidence of sEMR was reduced with trains of stimulation in both groups due to an inability to tolerate the stimulation. Threshold intensities varied across lower limb muscles, and medial hamstring muscles required the highest stimulation intensity to evoke sEMR. The incidence of sEMR was less in participants with a SCI for single pulses, but similar to non‐SCI participants for trains of stimulation.

### 4.1 Electrode configuration

The three electrode configurations used in the current study have been used previously (Dy et al., 2010; Hofstoetter et al., 2018; Hofstoetter, Krenn, et al., 2015; Islam et al., 2021; McHugh et al., 2020; Minassian et al., 2016). In non‐SCI participants, placing the anode over the ASIS was inferior to placing it midline over the lower abdomen because it led to lower sEMR incidence and higher threshold intensities. This may be due to the dispersion of the electrical field across the split anode and a potential increase in the distance from the cathode (Danner et al., 2011  Ladenbauer et al., 2010). Like the current findings, cervical spine stimulation evokes a sEMR with a lower intensity when the anode is located directly opposite the cathode compared to when the anode is split across two electrodes placed over the clavicles or the iliac crests (de Freitas et al., 2021). Together, these findings suggest that anode placement can impact the intensity of TSS required to evoke sEMR.

Because of the relatively clear results in non‐SCI participants, and because we wanted to decrease the burden on participants with a SCI, the L1‐ASIS configuration was not tested in participants with a SCI. When this decision was made, we saw no reason why the effect of anode location would differ between non‐SCI participants and participants with a SCI. While we still agree with this logic, including the L1‐ASIS configuration would have provided a more complete dataset and thus would have further strengthened the conclusions of the present study.

The T11‐midline and L1‐midline electrode configurations were largely comparable. The incidence of sEMR was 10% higher for the L1‐midline configuration compared to the T11‐midline electrode configuration, for both single pulses and trains of stimulation. Moreover, cathode placement changed the recruitment order of sEMR in lower limb muscles. Across the T11‐midline and L1‐midline electrode configurations, for both participant groups, VM was recruited first with the T11‐midline configuration. With the L1‐midline configuration, the MG muscle was generally recruited first. These results are consistent with those of previous studies (Krenn et al., 2013; Militskova et al., 2020; Roy et al., 2012; Salchow‐Hömmen et al., 2019; Sayenko et al., 2015) and are likely due to the proximity of the relevant spinal roots to the cathode. Overall, while the difference between the T11 and L1 cathode electrode placements was small in our study, the L1‐midline electrode configuration elicited sEMRs in more lower limb muscles for a given stimulation intensity, especially in people with a SCI where threshold intensities were not as divergent between these two electrode configurations as non‐SCI individuals. While previous studies, conducted in supine participants with small circular cathode electrodes over T11–T12, noted little to no difference in the order in which lower limb muscles were recruited (Hofstoetter et al., 2018; Minassian et al., 2007), we noted that the medial hamstring muscle was typically the last recruited muscle. Whether this difference in how the muscle with the most distal myotome (i.e., S2) is recruited is caused by the upright posture of our participants or the use of larger electrodes is unclear. Finally, in practical terms, being able to measure the sEMR threshold intensity in one muscle that represents the other lower limbs would be advantageous. Our data suggest that in participants with a SCI, using the L1‐midline configuration results in the VM sEMR threshold intensity ranking between the first and last lower limb muscles threshold intensities. Using the VM sEMR threshold would result in a balanced bias in stimulation intensity between the first and last recruited muscle, which contrasts with the other approach of using the intensity of the last recruited muscle (Minassian et al., 2016; Krenn et al., 2023).

### 4.2 Single pulses versus trains of stimulation

In non‐SCI participants, the stimulation threshold was 13% lower for trains of stimulation compared to single pulses. Nevertheless, the incidence of sEMR was 30%–50% lower in both participant groups with trains of stimulation. Across all muscles in which sEMR could not be evoked during trains of stimulation, the stimulation intensity that could be reached during trains of stimulation was 64% (SD 18; non‐SCI) and 76% (SD 15; SCI) of the threshold intensity with single pulses. This reflects an inability of participants to tolerate trains of stimulation at sufficiently high intensities to elicit sEMR.

Several studies set stimulation intensity for TSS therapy (i.e., trains) to 80%–100% of the threshold intensity obtained with single pulses (Hofstoetter et al., 2020; Meyer et al., 2020; Siu et al., 2022; Sutor et al., 2022; Zaaya et al., 2021). Some studies set the therapeutic intensity based on the threshold intensity to elicit sEMR in all muscles recorded (Minassian et al., 2016), or up to 130%–140% of sEMR threshold (Shapkova et al., 2020). Our participants frequently were not able to achieve these intensities due to discomfort. The highest intensity tolerated is usually the fall‐back criteria when paresthesia or sEMR thresholds cannot be elicited (Al'joboori et al., 2020; McHugh et al., 2020; Samejima et al., 2022; Zaaya et al., 2021). Our data indicate that when using the highest intensity tolerated, the values in relation to sEMR threshold vary widely between participants and muscles. This would often lead to the stimulation intensity being below 65%–75% of the single pulse stimulation threshold intensity (Figure 6b,f gray). Currently, the lower limit for effective stimulation intensity has not been established.

It is worth noting that an inability to tolerate trains of stimulation intensities at single‐pulse sEMR threshold intensity was shared between non‐SCI and participants with an SCI. Most of our participants had sensory incomplete lesions and were able to feel the stimulation. One participant with an injury classified as AIS‐A (T7 injury) was unable to tolerate trains of stimulation that evoked sEMR in any lower limb muscle with the T11‐midline electrode configuration. This was due to discomfort caused by contractions of the abdominal and paraspinal muscles, which was perceived by the participant to affect their breathing. Of interest, with trains of stimulation, we were only able to evoke sEMR in at least one muscle in three participants with a SCI using the T11‐midline configuration compared to six of the nine participants with a SCI using the L1‐midline configuration.

### 4.3 Possible mechanisms underlying differences in threshold intensity between single pulses and trains of stimulation

We hypothesized that the stimulation intensity required to elicit sEMR would be higher during trains of stimulation compared to single pulses. However, our results did not support this hypothesis. In fact, compared to single pulses, threshold intensity with trains of stimulation was unchanged in participants with a SCI and lower in non‐SCI participants. Several mechanisms that increase or decrease the excitability of motoneurones may be activated by trains of stimulation. For example, post‐activation depression (Hultborn et al., 1996; Lamy et al., 2005) and presynaptic inhibition (Eccles et al., 1962; Iles & Roberts, 1987) of the Ia afferents can be activated by trains of stimulation. However, both mechanisms are reduced in people with a SCI (Caron et al., 2020; Faist et al., 1994; Grey et al., 2008; Hofstoetter et al., 2019). While this could explain the lack of a difference in the threshold intensity between trains of stimulation and single pulses in the SCI group, it cannot explain the reduced threshold intensity in the non‐SCI group.

Other mechanisms that decrease the excitability of the neural pathway involved in sEMR generation are likely to have been activated by our trains of stimulation at 20 Hz. These include recurrent inhibition (Hofstoetter, Danner, et al., 2015), activation during the after‐hyperpolarization of the motoneurone (~100 ms; Christie & Kamen, 2010; Matthews, 1996), and prolonged hyperpolarization of dorsal root nerve fibers (Bostock et al., 1998; Burke et al., 2001). While these mechanisms should increase stimulation threshold intensity during trains of stimulation, this did not occur in either of our participant groups. However, consistent with past studies that explored stimulation frequencies effects (Jilge, Minassian, Rattay, Pinter, et al., 2004; Sayenko et al., 2019; Sharma & Shah, 2021; Vargas Luna et al., 2021), the amplitude of sEMR at threshold intensity during trains of stimulation was reduced compared to single pulses, a result that may reflect some or all of these mechanisms. A possible explanation for the lower recruitment threshold for sEMR in non‐SCI participants during trains of stimulation is the activation of excitatory interneuronal circuits that are not activated during single‐pulse stimulation (Guru et al., 1987; Jilge, Minassian, Rattay, & Dimitrijevic, 2004; Kathe et al., 2022). Activation of interneuronal circuits may also be responsible for the longer latencies of sEMR evoked during trains of stimulation compared to single pulses (Data S1 and S2), indicative of a longer reflex pathway (Jilge, Minassian, Rattay, & Dimitrijevic, 2004; Minassian et al., 2002, 2004). However, the current study did not show similar changes for the SCI group, including no change in the amplitude and latency of the sEMR at threshold intensity with trains of stimulation (cf Minassian et al., 2004).

### 4.4 Incidence of sEMR in participants with spinal cord injury

Despite both participant groups reaching similar stimulation intensities with single‐pulse TSS, the incidence of sEMR in participants with a SCI was about 30% lower than in non‐SCI participants. In some participants, sufficiently high‐stimulation intensities could not be tolerated, while in other participants sEMR could not be elicited despite large increases in stimulation intensity, which suggests they had higher threshold intensities (see Figure 4). Others have reported a similar inability to evoke a sEMR in some participants with a SCI (Hofstoetter et al., 2019; Meyer et al., 2020; Shapkova et al., 2020), with the causes unexplained. This implied increase in sEMR threshold intensity in participants with a SCI could be caused by differences in adipose tissue under the stimulation electrodes (Kuhn et al., 2009; Ladenbauer et al., 2010), although BMI was similar between groups. It could also reflect differences in body position as changes in spinal curvature or weight bearing through the lower limbs has been shown to influence sEMR stimulation intensity (Binder et al., 2021; Megía‐García et al., 2021; Militskova et al., 2020). In the present study, non‐SCI participants were suspended upright in a harness and participants with a SCI were titled to 55°. Although unlikely given that the curvature of the spine was similar in the two positions and that both groups of participants wore the same tight‐fitting harness, participant position may have influenced the incidence of sEMR in our study.

### 4.5 Limitations

As was just highlighted, we modified the upright position when we tested participants with a SCI. Although not optimal, this protocol deviation was introduced after four non‐SCI participants experienced hypotension. To reduce between‐group differences, participants with a SCI wore the body‐weight support harness, with the straps tightened in the same way as non‐SCI participants. The fact that participant position was not identical between the two participant groups should be kept in mind when interpreting between‐group differences presented in the present study.

Threshold intensities were determined at a resolution of 10 mA. This was the most efficient resolution to determine threshold intensities in several lower limb muscles, with two to three electrode configurations and two stimulation types (single pulses and trains). The selected step size is commensurate with the higher threshold intensities (Manson et al., 2020) and the higher spread of threshold intensities that occurs with kHz stimulation (see Figure 4). Our approach is not as accurate as individually determining the threshold intensity of all eight lower limb muscles. However, greater precision in these estimates would likely have little to no influence on the results of the present study.

As a final point, our sample size was relatively small. While some of the results from the present study are clear, a few of the reported effects were associated with a relatively large 95% CI. Thus, care must be taken when interpreting some of our results. Moreover, our results indicate that to obtain more precise estimates, future studies of the type conducted here should have a larger sample size.

## 5 CONCLUSIONS

For high‐frequency TSS, our findings suggest that the T11‐midline and L1‐midline electrode configurations evoke sEMR similarly with single pulses and trains of stimulation in non‐SCI participants and participants with a SCI. However, as outlined in the Discussion, we favor the use of the L1‐midline configuration because: (1) for both single pulses and trains of stimulation the incidence of sEMR was 10% higher, and (2) the number of participants with a SCI in whom sEMR could be evoked with trains of stimulation was twice as high. We also recommend the use of the threshold intensity for VM with single‐pulse TSS to set the therapeutic intensities for trains of stimulation because it represents the threshold intensity of several key muscles of locomotion; also, the VM has relatively larger sEMR amplitudes at threshold intensity enabling easier identification. Finally, contrary to expectation, the threshold intensity to recruit sEMR was not increased during trains of stimulation. Our results indicate that in people with SCI the therapeutic intensity for trains of stimulation can be based on the threshold intensity determined with single pulses. However, when individuals can tolerate only subthreshold stimulation intensities during trains of stimulation, knowing the single‐pulse threshold intensity allows investigators to determine the relative stimulation intensity of the trains of stimulation, which provides a quantitative method to compare stimulation intensity across participants and studies.

## AUTHOR CONTRIBUTIONS

Study design: Elizabeth A. Bye, Thomas G. Elphick, Simon C. Gandevia, Jane E. Butler, Martin E. Héroux; Data collection: Elizabeth A. Bye, Thomas G. Elphick, Martin E. Héroux; Data analysis: Harrison T. Finn, Martin E. Héroux; Writing—original draft: Harrison T. Finn, Jane E. Butler, Martin E. Héroux; Writing—review and editing: All authors; Project administration: Harrison T. Finn, Elizabeth A. Bye, Martin E. Héroux; Funding acquisition: Simon C. Gandevia and Jane E. Butler.

## FUNDING INFORMATION

Funding for this study has been received from SpinalCure Australia and Catwalk NZ. SCG and JEB are supported by the National Health and Medical Research Council of Australia (NHMRC).

## ETHICS STATEMENT

The procedures were approved by the University of New South Wales Human Research Ethics Committee (HC190731) and the study was conducted in accordance with the Declaration of Helsinki (except for pre‐registration in a database). All participants gave written informed consent.

## Supporting information


Data S1
