# Supplementary material for: Transcutaneous spinal stimulation in people with and without spinal cord injury: Effect of electrode placement and trains of stimulation on threshold intensity
Source: Physiol Rep. 2023 Jun 2;11(11):e15692. doi: 10.14814/phy2.15692 (PMC10238786; doi:10.14814/phy2.15692)
Supplement: Supplementary file 1 — Data S1 [file PHY2-11-e15692-s001.docx]

**ONLINE SUPPLEMENT 1 – Latency (milliseconds) of spinally evoked motor responses**

|  | non-SCI | | | | | | SCI | | | | |  |
| --- | --- | --- | --- | --- | --- | --- | --- | --- | --- | --- | --- | --- |
|  | Single | | | Train | | | Single | | Train | | |  |
|  | T11-midline | L1-ASIS | L1-midline | T11-midline | L1-ASIS | L1-midline | T11-midline | L1-midline | | T11-midline | L1-midline | |
| VM | 8.6 (1.4) | 9.9 (1.8) | 9.0 (1.4) | 10.2 (1.7) | 13.2 (1.8) | 10.9 (1.7) | 12.2 (4.1) | 11.0 (2.5) | | 11.0 (2.4) | 11.8 (2.2) | |
| Ham | 12.1 (3.6) | 10.6 (2.5) | 10.8 (3.0) | 9.7 (3.7) | 14.6 (4.1) | 11.0 (1.8) | 14.1 (3.9) | 11.8 (3.5) | | 12.0 (3.0) | 11.6 (1.5) | |
| MG | 15.7 (2.8) | 16.0 (2.8) | 15.6 (2.8) | 18.3 (1.9) | 16.3 (2.4) | 17.2 (2.8) | 16.4 (2.0) | 17.5 (2.3) | | 16.8 (2.1) | 15.6 (3.4) | |
| TA | 14.1 (2.7) | 15.0 (2.5) | 14.1 (2.7) | 16.5 (1.9) | 17.1 (2.9) | 16.2 (3.1) | 17.2 (3.8) | 17.5 (2.4) | | 16.9 (2.5) | 18.0 (2.1) | |

Values are mean (SD). For the non-spinal cord injury (non-SCI) participants, there was an effect of stimulation type (F_(1, 247)_=14.204, p<0.001). The single pulses produced spinally evoked motor response (sEMR) at a shorter latency than the trains of stimulation(p<0.001), with the average difference being 1.40 ms [0.67 to 2.14]. There was also a main effect of electrode configuration (F_(2, 247)_=3.99, p=0.020). The L1-ASIS configuration evoked sEMR with longer latencies compared to the L1-midline configuration (mean difference 1.29 ms [0.16 to 2.41], p=0.019), but latencies were not different to those recorded using the T11-midline configuration (mean difference 0.94 ms [-0.08 to 1.95], p=0.076). T11-midline and L1-midline configurations were not different (mean difference 0.35 ms [-0.47 to 1.16], p=0.42). As expected there was a main effect of muscle group (F_(3, 247)_=74.567, p<0.001) with the latency for the sEMR onset in the vastus medialis muscle (VM) being significantly shorter than in the medial hamstring muscle (Ham) (mean difference -1.24 ms [-2.4 to -0.07], p=0.035), tibialis anterior muscle (TA) (mean difference -5.37 ms [-6.66 to -4.08], p<0.001) and medial gastrocnemius muscle (MG) (mean difference -6.28 ms [-7.58 to -4.98], p<0.001).

However for the participants with a SCI, there was no effect of electrode configuration (F_(1, 126)_=1.494, p=0.224), or type of stimulation (F_(1, 126)_=1.382, p=0.242). Individual muscles again had different latencies with an effect of muscle group on sEMR latency (F_(3, 126)_ = 65.98, p<0.001) and followed the same order of latencies as for the non-SCI participants. The latency in VM was shorter than in Ham (mean difference -1.55 ms [-2.92 to -0.18], p=0.023), MG (mean difference -5.57 ms [-6.94 to -4.2], p<0.001) and TA (mean difference -6.29 ms [-7.68 to -4.9], p<0.001).

When comparing the latencies between the participant groups, there was an effect of participant group (F_(1, 320)_=8.631, p=0.004). The sEMR had a longer latency in the SCI group overall by 2.57 ms [0.85 to 4.29] (p=0.004). There was no interaction effect between participant group and electrode configuration (F_(1, 320)_=0.114, p=0.736), participant group and muscle group (F_(3, 320)_=2.529, p=0.057) and participant group and type of stimulation (F_(1, 320)_=0.56, p=0.455).

**ONLINE SUPPLEMENT 2 – Amplitude (millivolts) of spinally evoked motor responses**

|  | non-SCI | | | | | | SCI | | | | |
| --- | --- | --- | --- | --- | --- | --- | --- | --- | --- | --- | --- |
|  | Single | | | Train | | | Single | | | Train | |
|  | T11-midline | L1-ASIS | L1-midline | T11-midline | L1-ASIS | L1-midline | T11-midline | L1-midline | T11-midline | | L1-midline |
| VM | 0.039 (0.032) | 0.055 (0.115) | 0.047 (0.069) | 0.056 (0.076) | 0.005 (0.001) | 0.014 (0.011) | 0.034 (0.027) | 0.05 (0.053) | 0.019 (0.013) | | 0.019 (0.015) |
| Ham | 0.02 (0.006) | 0.027 (0.027) | 0.028 (0.022) | 0.006 (0.002) | 0.005 (0.002) | 0.008 (0.002) | 0.029 (0.014) | 0.042 (0.046) | 0.013 (0.002) | | 0.011 (0.003) |
| MG | 0.044 (0.037) | 0.028 (0.015) | 0.036 (0.027) | 0.008 (0.003) | 0.006 (0.002) | 0.014 (0.01) | 0.034 (0.025) | 0.032 (0.018) | 0.008 (0.001) | | 0.009 (0.004) |
| TA | 0.023 (0.007) | 0.02 (0.005) | 0.021 (0.008) | 0.011 (0.008) | 0.011 (0.009) | 0.013 (0.008) | 0.023 (0.014) | 0.034 (0.038) | 0.01 (0.002) | | 0.009 (0.003) |

Values are mean (SD). For non-spinal cord injury (non-SCI) participants, the amplitude of the spinally evoked motor responses (sEMR) showed an effect of muscle group (F_(3, 253)_=4.446, p=0.005). The sEMR in the vastus medialis muscle (VM) had the largest amplitudes at threshold as compared to the medial hamstring muscle (Ham) (mean difference 0.026 mV [0.004 to 0.047], p=0.01) and the tibialis anterior muscle (TA) (mean difference 0.024 mV [0.004 to 0.045], p=0.013), but not the medial gastrocnemius muscle (MG) (mean difference 0.018 mV [-0.001 to 0.038], p=0.08). The amplitude of the sEMR in Ham, TA and MG were not different from each other (p>0.9). There was an effect of type of stimulation (F_(1, 253)_=8.52, p=0.004) with the train stimulation resulting in smaller sEMR at threshold (mean difference -0.018 mV [-0.03 to ­0.006], p=0.004) compared to the sEMR elicited by single pulses. There was no effect of stimulation configuration (F_(2, 253)_=0.232, p=0.793).

For the participants with a SCI, the amplitude of the sEMR showed an effect of stimulation type (F_(1, 129)_=19.901, p<0.001). The sEMR was smaller for the train stimulation as compared to single pulses (mean difference -0.022 mV [-0.031 to -0.012], p<0.001). There was no effect of muscle (F_(3, 129)_=2.203, p=0.091) or electrode configuration (F_(1, 129)_=2.605, p=0.109) on the amplitude of the sEMR.
